# Adaptive Autoencoder-Based Intrusion Detection System with Single Threshold for CAN Networks

**DOI:** 10.3390/s25134174

**Published:** 2025-07-04

**Authors:** Donghyeon Kim, Hyungchul Im, Seongsoo Lee

**Affiliations:** Department of Intelligent Semiconductors, Soongsil University, Seoul 06978, Republic of Korea; aflolk97@soongsil.ac.kr (D.K.); tory@soongsil.ac.kr (H.I.)

**Keywords:** controller area network, cybersecurity, intrusion detection system, in-vehicle network, deep learning, Gaussian kernel density estimation, lightweight

## Abstract

The controller area network (CAN) protocol, widely used for in-vehicle communication, lacks built-in security features and is inherently vulnerable to various attacks. Numerous attack techniques against CAN have been reported, leading to intrusion detection systems (IDSs) tailored for in-vehicle networks. In this study, we propose a novel lightweight unsupervised IDS for CAN networks, designed for real-time, on-device implementation. The proposed autoencoder model was trained exclusively on normal data. A portion of the attack data was utilized to determine the optimal detection threshold using a Gaussian kernel density estimation function, while the frame count was selected based on error rate analysis. Subsequently, the model was evaluated using four types of attack data that were not seen during training. Notably, the model employs a single threshold across all attack types, enabling detection using a single model. Furthermore, the designed software model was optimized for hardware implementation and validated on an FPGA under a real-time CAN communication environment. When evaluated, the proposed system achieved an average accuracy of 99.2%, precision of 99.2%, recall of 99.1%, and F1-score of 99.2%. Furthermore, compared to existing FPGA-based IDS models, our model reduced the usage of LUTs, flip-flops, and power by average factors of 1/5, 1/6, and 1/11.

## 1. Introduction

In the past, the relatively low sophistication of hacking techniques meant that the security of in-vehicle networks did not attract significant attention. However, as these techniques have advanced and attack methods have diversified, incidents have emerged in which vulnerabilities in in-vehicle networks were exploited to cause vehicle malfunctions [1]. As a result, even conventional vehicle systems are increasingly recognizing the importance of securing internal networks. Moreover, the rise of autonomous vehicles has further heightened concerns regarding automotive cybersecurity.

**Figure 1:**
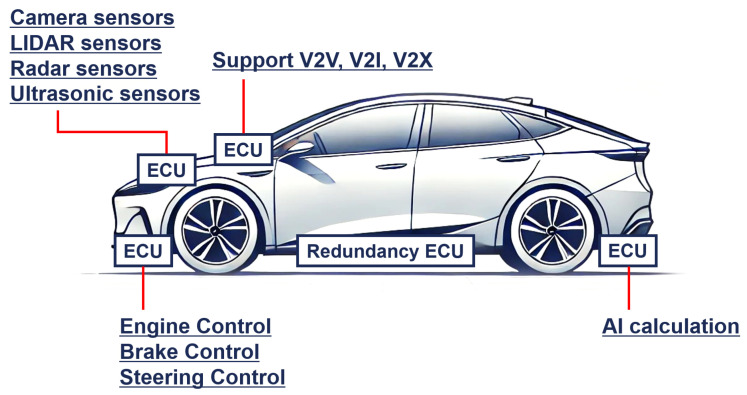
Interactions between various sensors, control systems, and ECUs employed in autonomous vehicles.

Autonomous driving systems employ a multitude of integrated sensors, including cameras, light detection and ranging (LiDAR), radar, and ultrasonic sensors, to detect and analyze the surrounding environment in real time. As the complexity of autonomous driving technology increases, traditional control systems, such as engines, brakes, and steering controls, are becoming more advanced. Consequently, the number of ECUs managing these sensors and systems has increased. Furthermore, autonomous vehicles utilize artificial intelligence and machine learning-based algorithms to process and analyze various real-time scenarios encountered during driving. This growing computational demand has led to an increasing need for dedicated ECUs for handling advanced operations. Finally, autonomous vehicles exchange information with external entities through vehicle-to-vehicle (V2V), vehicle-to-infrastructure (V2I), and vehicle-to-everything (V2X) communication systems, which require independent ECUs to support the corresponding connectivity modules. The interactions among the various sensors, control systems, and ECUs employed in autonomous vehicles are illustrated in Figure 1.

Thus, the progression of autonomous driving technology has significantly increased the demand for ECUs in vehicles, resulting in increased electronic and communication complexities across the entire vehicle system. The proliferation of ECUs has intensified the frequency of data exchange within in-vehicle networks, elevating network security to a critical level. Because each ECU exchanges data with various sensors and systems in real time and plays a vital role in vehicle control and operation, exposure to external attacks poses severe security risks. As autonomous vehicles enhance their connectivity with road environments and external systems, the development of robust security technologies to safeguard in-vehicle networks has become increasingly crucial [2,3,4].

The core of the vehicle network is the controller area network (CAN), which manages data transmission and receipt between the ECUs. The CAN protocol enables efficient communication between various electronic systems within a vehicle and provides flexibility through its multimaster architecture, allowing any node to initiate data transmission. However, the CAN does not assign specific addresses to transmitting or receiving nodes and lacks encryption or other security features, making it inherently vulnerable to attacks.

Various intrusion detection systems (IDSs) have been proposed to address these weaknesses in CAN communication. Machine learning-based approaches, such as the use of support vector machines (SVMs) to detect normal and attack frames [5] and the nearest neighbor algorithm [6], have been introduced. Deep learning methods have also been proposed for CAN attack detection [7]. However, supervised learning-based IDSs are only effective in detecting known attack types and fail to identify novel attack patterns. To overcome this limitation, unsupervised learning-based IDSs, such as those employing generative adversarial networks (GANs) [8] and isolation forests (iForests) [9], have been proposed. Despite their ability to detect unknown attacks, these models generally exhibit lower performance than supervised learning-based IDSs. NovelADS, another unsupervised IDS [10], achieves excellent performance but requires different thresholds for each attack type, which is a notable drawback. In addition to software-based approaches, hardware-oriented CAN IDSs have also been proposed. For example, an IDS utilizing a Quantized Multi-Layer Perceptron (QMLP) was proposed and validated on a ZCU104 board [11]. Another IDS based on a Binarized Neural Network (BNN) was proposed and tested using a ZedBoard [12]. While these FPGA-based implementations demonstrated the feasibility of deploying CAN IDSs in hardware, they suffer from significant hardware resource consumption. Table 1 summarizes the limitations of various existing CAN IDSs and illustrates how the proposed model addresses these challenges.

In addition to IDSs applied to CAN communication, various IDSs have also been proposed in other domains. In particular, several IDSs based on autoencoder models have been proposed [13]. For example, [14] combined convolutional neural networks (CNNs) and long short-term memory (LSTM) networks as encoder and decoder layers of an autoencoder-based IDS. Similarly, [15,16] proposed IDSs that use LSTM-based autoencoders. In [17], an IDS that combines sparse regularization convolutional autoencoders (SRCAEs) with a stream-clustering model was introduced, whereas [18] employed an autoencoder with an attention mechanism. Furthermore, [19] proposed an IDS based on deep contractive autoencoders (DCAEs), and [20] applied a gated recurrent unit (GRU)-based autoencoder. In addition, IDSs based on deep learning models have also been proposed. The authors of [21] presented various IDSs that leverage Deep Reinforcement Learning (DRL) across multiple application domains. Similarly, [22] introduced IDSs based on Deep Transfer Learning (DTL) applied in diverse fields.

In this study, we propose an autoencoder-based IDS that determines the optimal frame count and threshold using a Gaussian kernel density estimation (KDE) function. The proposed model was built using an unsupervised learning approach that was initially trained solely on normal CAN traffic. The proposed model adopts a simple autoencoder architecture consisting of a single flatten layer, two dense layers, and one reshape layer. Subsequently, it effectively detected attacks by analyzing CAN traffic containing malicious data. The simulation results demonstrate that the proposed model outperforms existing unsupervised learning-based IDSs. Finally, the software-verified model was compressed for lightweight implementation and designed for hardware deployment. The resulting model was then integrated with an ARM Cortex-M3 processor and a CAN controller on the Nexys Video FPGA board for validation.

The main contributions of this study are as follows.

1. This paper presents the development of a deep learning-based IDS for in-vehicle networks. To effectively detect unknown attacks, an unsupervised learning model, specifically an autoencoder, was employed.
2. The proposed model requires the determination of the optimal number of data frames to be grouped during training and the threshold for distinguishing between normal and attack data to achieve high performance. A KDE function was utilized to identify the optimal frame count and threshold.
3. The IDS model, initially validated in software, was redesigned as a lightweight hardware implementation. It was deployed on an FPGA board and evaluated under real-time CAN communication.

The remainder of this paper is organized as follows. Section 2 presents the theoretical background, including an overview of the CAN protocol, CAN bus attacks, the autoencoder model, and the KDE function. Section 3 describes the proposed system in detail, including the dataset, data preprocessing, model structure, and the method for determining the optimal threshold and frame count. In addition, the software-based model is optimized into a lightweight hardware implementation, and the verification process is described along with the corresponding evaluation environment. Section 4 reports the experimental results along with performance metrics, discussion, and limitations. Finally, Section 5 concludes the paper and outlines directions for future research.

## 2. Theoretical Background

In this section, we provide an overview of CAN protocols, discuss various attack methods targeting CAN buses, and introduce the autoencoder model and its relevance to the proposed approach. Finally, we elaborate on the KDE function and its applications in the context of this study.

### 2.1. Controller Area Network

#### 2.1.1. Controller Area Network Overview

**Figure 2:**
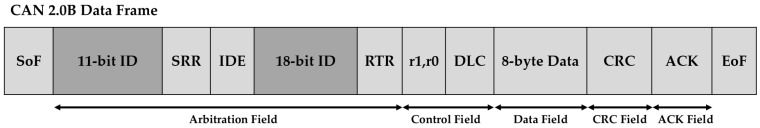
CAN 2.0B data frame format.

The CAN protocol was developed by Bosch in the 1980s to facilitate efficient communication between various ECUs within a vehicle [23]. Subsequently, it was adopted as an international standard (ISO 11898) in 1993 and has since been widely utilized not only in vehicles but also in other fields such as aviation and medical devices. The CAN is a multimaster message-based network that allows multiple nodes to communicate over a shared bus. All nodes in the network share the same transmission medium, and any node requiring communication autonomously transmits data. Each data packet is transmitted with a specific ID indicating the priority of the data. This ID is also employed to prevent collisions when multiple messages are transmitted simultaneously in a network. In such cases, messages with higher-priority IDs are transmitted first, and lower-priority messages attempt retransmission. CANs can be categorized into two types. The first is a standard CAN (CAN 2.0A), which uses an 11-bit ID to distinguish up to 2048 messages. The second is an extended CAN (CAN 2.0B), which employs a 29-bit ID, enabling the identification of a greater number of messages. Figure 2 illustrates the CAN 2.0B data frame format.

The CAN 2.0B data frame consists of an 11-bit ID and 18-bit extended ID, along with several fields and single-bit components. The primary fields include arbitration, control, data, CRC, and acknowledgment (ACK) fields. A data frame begins with a single-bit start of frame (SoF) and ends with a single-bit end of frame (EoF). Detailed descriptions of each field and single-bit component are provided below.

- **Start of Frame:** The SoF is a single bit that marks the beginning of the frame, indicating the initiation of communication.
- **Arbitration field:** The arbitration field consists of an 11-bit standard ID, a 1-bit substitute remote request (SRR), a 1-bit identifier extension (IDE), an 18-bit extended ID, and a 1-bit remote transmission request (RTR). This field determines the priority of the message. The SRR bit ensures compatibility between CAN 2.0A, which uses an 11-bit ID, and CAN 2.0B, which employs a 29-bit ID. The IDE bit distinguishes between CAN2.0A and CAN2.0B, while the RTR bit differentiates between data frames and remote frames.
- **Control field:** The control field is composed of r1, r0, and the data length code (DLC), which define the message format and data length. The r1 and r0 bits are reserved, while the DLC specifies the size of the data field.
- **Data field:** The data field contains the actual data being transmitted and allows for a maximum data size of up to 8 bytes.
- **CRC field:** The CRC field ensures the integrity of the transmitted data and is used for error detection.
- **ACK field:** The ACK field is an acknowledgment bit that indicates successful receipt of the message by the receiver.
- **End of Frame:** The EoF is a single bit that marks the conclusion of the frame and signals the completion of transmission.

#### 2.1.2. CAN Bus Attack

The CAN bus refers to the network in which the CAN protocol is implemented, which facilitates communication between multiple control units or nodes interconnected via the CAN. The CAN bus adopts a bus-type network structure in which all the nodes are connected to the same physical bus and communicate in parallel with other nodes in the network. This design enables efficient and low-cost communication among devices, while offering excellent scalability, because new nodes can be easily added.

However, because every node in the CAN bus can act as a master node and there is no authentication for data frame transmission, the system is inherently vulnerable to security breaches [24,25,26]. To exploit this vulnerability, recent studies have proposed various CAN bus attack methods. One study demonstrated that by using an on-board diagnostics (OBD)-II port, an attacker can disable braking systems or cause a sudden increase in the RPM during vehicle operation [1]. Another study revealed that attacks can be conducted remotely using a wireless OBD-II dongle [27].

Representative types of CAN bus attacks include denial-of-service (DoS), fuzzy, and spoofing attacks. These attacks are frequently used in various studies because they can be reliably reproduced, public datasets are available, and they are difficult to detect in actual CAN communication. In DoS attacks, the same ID is repeatedly injected, making it hard to distinguish from burst mode behavior in normal traffic. Fuzzy attacks randomly inject a variety of IDs, and if an injected ID coincidentally matches one used in normal traffic, detection becomes challenging. Spoofing attacks mimic legitimate messages, making them difficult to detect using simple anomaly detection algorithms.

**Figure 3:**
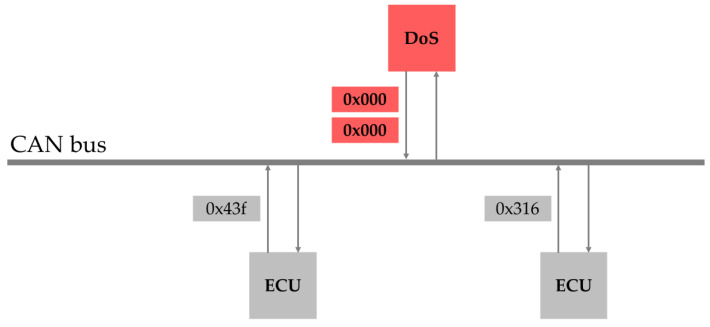
Illustration of a DoS attack.

A DoS attack involves the repeated injection of CAN frames with intentionally high-priority IDs into a CAN bus. This exploits the characteristics of CAN, where lower-priority IDs are ignored in the presence of higher-priority IDs, thereby continuously occupying the CAN bus. Consequently, normal nodes are unable to transmit or receive data, leading to paralysis of the CAN bus. For example, if an ID such as 0 × 000 is repeatedly injected, the CAN bus prioritizes this ID, which blocks the transmission and receipt of lower-priority IDs, such as 0 × 43f and 0 × 316, as shown in Figure 3.

**Figure 4:**
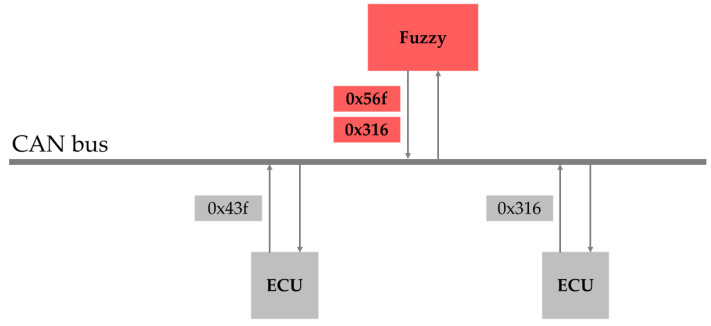
Illustration of a fuzzy attack.

A fuzzy attack involves random generation and injection of IDs and data into the CAN bus. If an ID actually used by the vehicle is injected during this process, it may cause a malfunction in the corresponding device. Additionally, the random occupation of the CAN bus interferes with the normal transmission and receipt of data between the nodes. For example, if the attack node randomly injects IDs and injects an ID such as 0 × 316, which is already in use, the functionality associated with this ID may malfunction, as shown in Figure 4.

**Figure 5:**
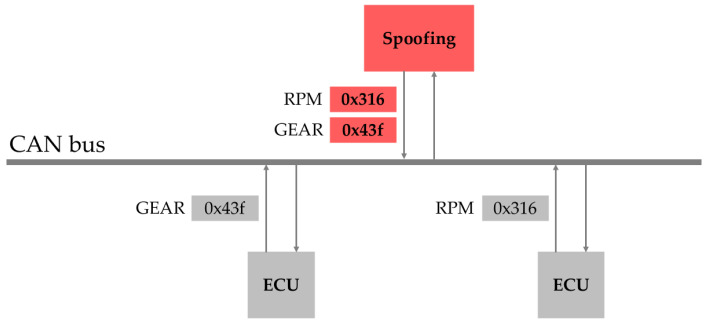
Illustration of a spoofing attack.

A spoofing attack involves analyzing and reversing the CAN traffic to identify IDs related to specific devices, such as RPM or gear settings, and then injecting these IDs into the CAN bus. Unlike DoS attacks, spoofing attacks inject IDs that have already been transmitted within the CAN bus, intentionally causing the vehicle to malfunction. An example of this type of attack is shown in Figure 5. In this case, IDs such as 0 × 316 and 0 × 43f, which correspond to the actual RPM functionality, were injected, resulting in a malfunction of the related features.

### 2.2. Autoencoder

**Figure 6:**
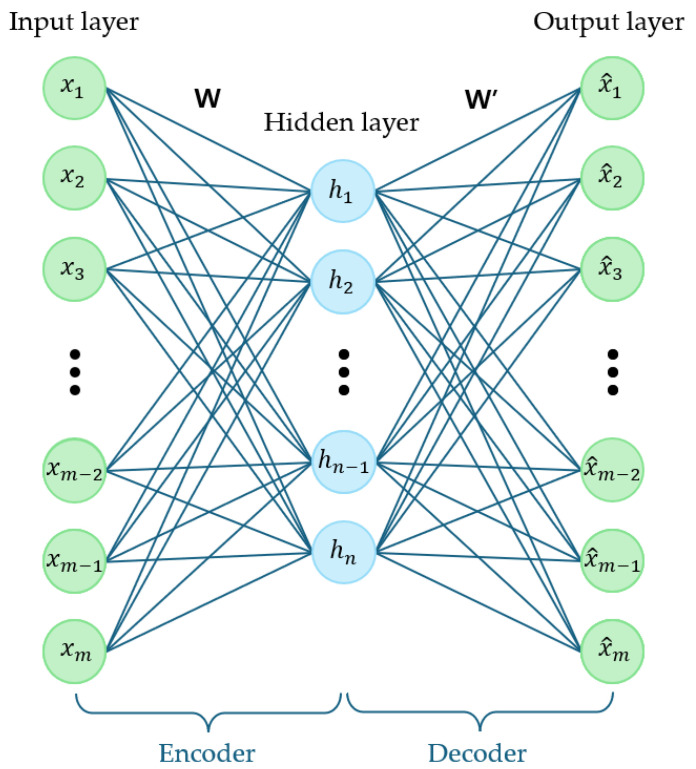
Illustration of a simple autoencoder.

An autoencoder is a neural network structure designed to compress the input data into a lower-dimensional space and then reconstruct it. A simplified representation of this architecture is presented in Figure 6.

This model comprises two main components: an encoder and a decoder. The encoder transforms high-dimensional input data into a lower-dimensional latent space, referred to as the hidden layer, and is expressed as follows:

(1) h=s(Wx+b),

where *h* represents the hidden vector, which belongs to ${\left[0,1\right]}^{n}$. *x* represents the input vector, which belongs to ${\left[0,1\right]}^{m}$. At this stage, *W* is an m×n weight matrix and *b* is a bias vector. s(x) denotes the activation function.

Similarly, the decoder reconstructs the latent representation back into the original dimension and is expressed as follows:

(2) $$\hat{x}=s\left(W^{\prime }h+b^{\prime }\right),$$

where $\hat{x}$ represents the restored vector, which belongs to ${\left[0,1\right]}^{m}$. At this stage, $W^{\prime }$ is an n×m weight matrix and $b^{\prime }$ is a bias vector.

The autoencoder focuses on extracting significant features from the input data and utilizing them for reconstruction. One of the primary applications of autoencoders is dimensionality reduction. Converting high-dimensional data into a lower-dimensional representation while minimizing the information loss facilitates data visualization and efficient processing [28]. Additionally, autoencoders can be employed for noise removal by estimating the original data from noisy inputs [29]. Furthermore, based on the characteristic that normal data are effectively reconstructed while anomalous data are not, autoencoders are widely used for anomaly detection.

### 2.3. Gaussian Kernel Density Estimation

The Gaussian KDE is a nonparametric method used to estimate the probability density function of a given dataset. Unlike parametric methods, KDE does not assume a specific data distribution. Instead, it estimates density by smoothly approximating the region around each data point, resulting in a probability density function. The Gaussian kernel is one of the most commonly used kernels in KDE and applies a kernel with the shape of a Gaussian normal distribution centered at each data point. The KDE function is expressed as follows:

(3) $$f\left(x\right)=\frac{1}{nh}\sum_{i=1}^{n}K\left(\frac{x-x_{i}}{h}\right),$$

where *n* represents the number of samples, and *h* is a value referred to as the bandwidth that controls the width of the kernel. K(x) denotes the kernel function; in the case of a Gaussian kernel, it takes the following form:

(4) $$K\left(x\right)=\frac{1}{\sqrt{2\pi }}exp\left(-\frac{x^{2}}{2}\right)$$

In KDE, the Gaussian kernel smoothly estimates the probability density around each data point based on the shape of the Gaussian distribution. The smoothness of the estimated density is determined by the value of *h*, which is known as the bandwidth. A larger bandwidth results in a smoother estimated distribution; however, if the bandwidth is excessively large, significant patterns in the data may be lost. Conversely, a bandwidth that is too small may lead to overly detailed patterns, thereby causing overfitting. The flexibility of KDE is particularly advantageous because of its nonparametric nature. Because it does not assume that the data follow a specific probability distribution, it can be adapted to various data distribution shapes. This makes it highly effective for estimating the probability densities in complex datasets.

## 3. Materials and Methods

This paper proposes a model that effectively detects attacks by training exclusively on preprocessed normal data without incorporating attack data during the training phase. Consequently, the model adopted an unsupervised learning approach rather than traditional supervised learning methods. In addition, the designed software model was converted into a lightweight hardware implementation, and a verification environment was constructed to evaluate its performance under real-time CAN communication.

### 3.1. Dataset

In this study, the vehicle hacking dataset provided by the Hacking and Countermeasure Research Lab [30] was used. This dataset was collected by connecting a Y-cable to the OBD-II port located beneath the steering system of the Hyundai YF Sonata vehicle. The training dataset consisted solely of normal CAN frames extracted from the dataset. For threshold determination, two-thirds of the data from four distinct attack scenarios—DoS, gear spoofing, RPM spoofing, and fuzzy attack—were utilized. The remaining attack data, which was not involved in the threshold selection process, was reserved as the test dataset for performance evaluation. The composition of this dataset is presented in Table 2.

The DoS attack involved injecting the 0 × 000 CAN ID every 0.3 ms. The fuzzy attack randomly injected CAN IDs and data every 0.5 ms. Spoofing attacks injected messages related to the RPM or gear every 1 ms. As shown in Table 2, all four attack datasets contain both attack and normal frames. These datasets were preprocessed using the zero-padding technique to create data of size *N* × 29 for validation.

### 3.2. Data Preprocessing

For training, we used only the CAN ID extracted from normal CAN data without attacks. Because CAN data frames support up to 29 bits, IDs with fewer than 29 bits were expanded to 29 bits using the zero-padding technique. Zero padding is a widely used method that appends zeros to the beginning or end of data to extend its size or standardize its format. Therefore, 11-bit CAN IDs were expanded to 29 bits using zero padding to ensure compatibility with both CAN 2.0A and CAN 2.0B. Subsequently, these CAN IDs were grouped into sets of *N* frames, forming two-dimensional data of size (*N*, 29) for training. This approach leverages the fact that the order in which the CAN IDs appear on the bus follows a consistent pattern, which allowed us to use these sequences for training. The value of *N* was set from 15 to 64 and 50 different training configurations were generated to determine the optimal *N* value. A detailed description of this process is provided in the following subsections.

### 3.3. Model Structure

**Figure 7:**
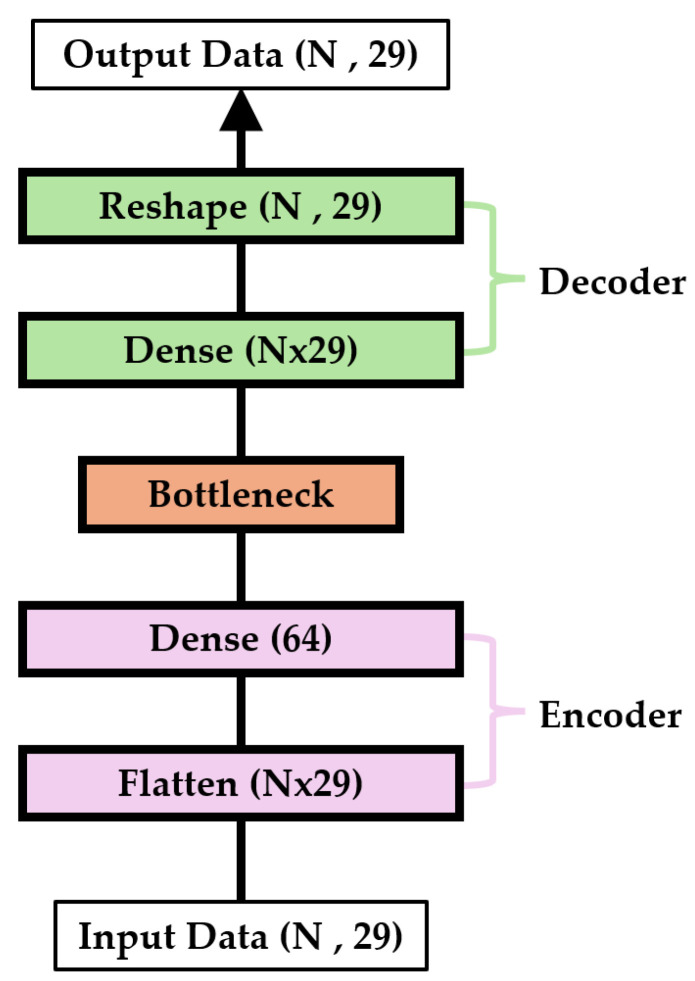
The structure of the proposed autoencoder model.

In this study, an autoencoder model was employed. Frequency-based filtering methods are effective in detecting attacks that cause significant changes in the frequency of specific CAN IDs, such as DoS attacks. However, they are limited in detecting attacks that do not substantially alter frequency patterns, such as fuzzy and spoofing attacks. Therefore, we employed an autoencoder, which can detect not only simple frequency anomalies but also subtle deviations within learned sequences. In addition, this model compresses input data into a low-dimensional latent space and subsequently reconstructs it. As a result, it produces low reconstruction error for normal data while generating higher error for malicious inputs. Due to this structural property, the model is considered well-suited for CAN intrusion detection. The encoder first receives the input data and compresses them into a lower-dimensional latent space using fully connected layers. Conversely, the decoder reconstructs the data back to their original form from this latent space using fully connected layers. The structure of the proposed autoencoder model used in this study is illustrated in Figure 7.

The encoder consists of a single flattened layer and a dense layer. The flattened layer receives two-dimensional input data of size (*N*, 29) and outputs *N* × 29 units. The dense layer in the encoder compresses the *N*× 29 units into 64 units. During this process, a rectified linear unit (ReLU) activation function is applied to perform nonlinear transformations. The decoder is composed of dense and reshaped layers. The dense layer of the decoder takes a compressed 64-unit representation as input and outputs *N* × 29 units. This dense layer uses a sigmoid activation function to restore the data. Finally, the reshaping layer receives *N* × 29 units and outputs the data in its original two-dimensional form of size (*N*, 29). The mean squared error (MSE) was selected as the loss function for the autoencoder. MSE is effective in quantifying the difference between the input and reconstructed data. Because the reconstruction accuracy is critical for autoencoders, MSE is a suitable loss function for this purpose and is expressed as follows:

(5) $$\mathrm{MSE}=\frac{1}{n_{m}}\sum_{i=1}^{n_{m}}{\left(y_{i}-\hat{y_{i}}\right)}^{2},$$

where $y_{i}$ represents the actual value, $\hat{y_{i}}$ is the predicted value, and $n_{m}$ is the total number of samples. Equation (5) allows an accurate evaluation of the model performance and optimization of the encoding and decoding processes of the autoencoder. To optimize the proposed autoencoder model, the learning rate was set to 0.001, and the optimizer was configured as an adaptive moment estimator (Adam). The Adam optimizer is widely used owing to its computational efficiency and excellent convergence properties. These settings played a critical role in optimizing the training process and effectively enhancing the performance of the model.

### 3.4. Determination of Optimal Threshold and Frame Count

The process of determining the optimal threshold and frame count for effective attack detection is described in Algorithm 1.
**Algorithm 1** Determination of Optimal Value of *N* and Threshold1: **Input:** Trained autoencoder model using *N* frames
2: **Output:** $N_{\mathrm{opt}}$ and $Th_{\mathrm{opt}}$
3: Initialize Y←∅, L←∅, E←∅
4: Initialize Attack types = (DoS,Fuzzy,RPM,Gear)
5: Initialize *k* = n(Attacktypes)
6: **for** *N* from 15 to 64 **do**
7:    $G_{1}$(*x*) = $KDE_{normal}$(*x*)
8:    **for** AT from Attacktypes **do**
9:      $G_{2}$(*x*) = $KDE_{AT}$(*x*)
10:      $Th_{AT}$ = $argmin_{x}$(|$G_{1}$(*x*) - $G_{2}$(*x*)|)
11:
12:      $ERE_{AT}=\frac{\int_{Th_{AT}}^{\infty }G_{1}\left(x\right)\;dx}{\int_{-\infty }^{\infty }G_{1}\left(x\right)\;dx}+\frac{\int_{-\infty }^{Th_{AT}}G_{2}\left(x\right)\;dx}{\int_{-\infty }^{\infty }G_{2}\left(x\right)\;dx}$
13:
14:    **end for**
15:
16:    $ERE_{avg}=\frac{ERE_{DoS}+ERE_{Fuzzy}+ERE_{RPM}+ERE_{Gear}}{k}$
17:
18:    **if** $Th_{DoS}=Th_{Fuzzy}=Th_{RPM}=Th_{Gear}$ **then**
19:      $Th_{eq}$ = $Th_{DoS}$
20:      Add *N* to set *Y*
21:      Add $Th_{eq}$ to set *L*
22:      $\mathrm{Add}\;ERE_{avg}\;\mathrm{to}\;\mathrm{set}\;E$
23:    **end if**
24: **end for**
25: Find $N_{opt}$ as the *N* with the smallest $ERE_{avg}$
26: Retrieve corresponding $Th_{eq}$ from *L* for $N_{opt}$, denote as $Th_{opt}$


In this study, the threshold was established based on the loss values obtained from the autoencoder. This threshold is closely related to the number of CAN frames (*N*) used during data preprocessing. To represent the distribution of loss values produced when attack data are input into the trained autoencoder, the Gaussian kernel density estimation (Gaussian KDE) function was employed. This function is a non-parametric method that does not assume a specific underlying distribution but instead estimates the density of loss values smoothly based on empirical data. Such an approach effectively captures the statistical differences across various types of attacks and enables anomaly detection using a single threshold. Due to these characteristics, it was considered well-suited for the proposed IDS. In this paper, Gaussian kernel density estimation is denoted as KDE and is expressed as follows:

(6) $$KDE\left(x\right)=\frac{1}{n_{k}h}\sum_{i=1}^{n_{k}}K_{g}\left(\frac{x-x_{i}}{h}\right),$$

where $K_{g}$ represents the Gaussian kernel function, $n_{k}$ denotes the number of loss values corresponding to either normal or attack data, *h* is the bandwidth, and $x_{i}$ represents the loss value.

**Figure 8:**
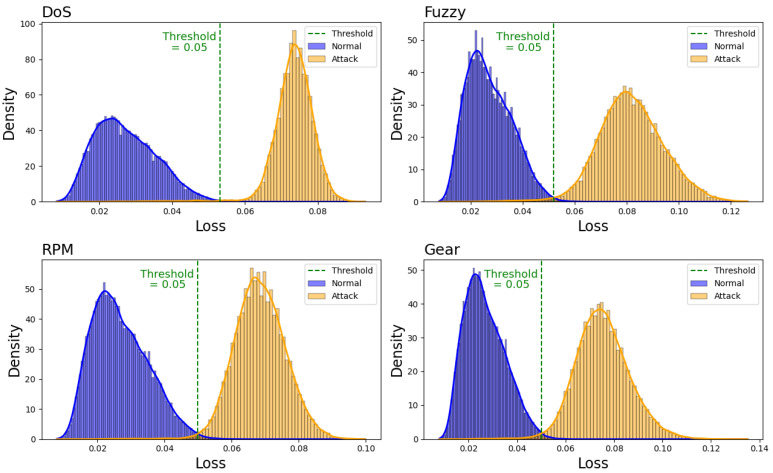
Distribution of autoencoder loss by attack type using the KDE function with *N* value of 40.

Using Equation (6), the distribution of the loss values for the four attack datasets was calculated when *N* = 40. The resulting distributions are presented in Figure 8.

**Figure 9:**
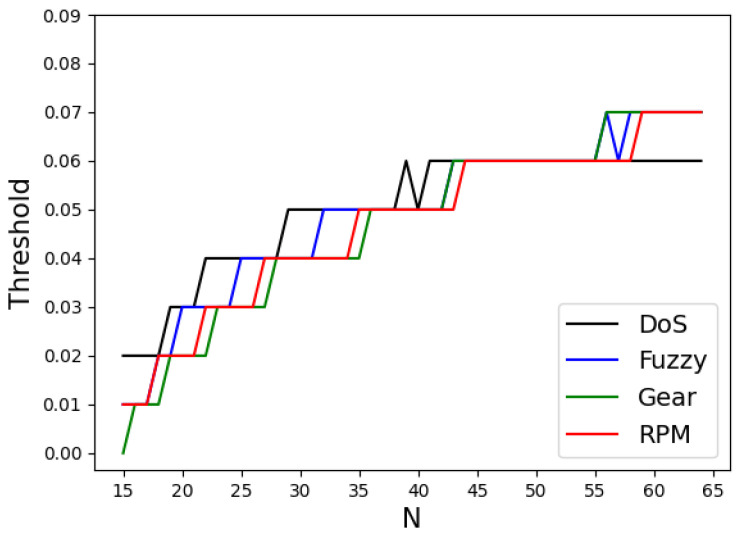
Threshold by attack type according to *N*.

In this context, “normal” refers to instances where none of the *N* CAN frames contain any attack frames, while “Attack” refers to cases where at least one attack frame is included among the *N* CAN frames. The threshold for distinguishing between attack and normal instances in this study was determined as the intersection point of the loss value distribution graphs for normal and attack data. Specifically, the threshold is defined as the loss value at which the two KDE functions intersect and is expressed as follows:

(7) $$Threshold=argmin_{x}\left|KDE_{0}\left(x\right)-KDE_{1}\left(x\right)\right|,$$

where $KDE_{0}\left(x\right)$ and $KDE_{1}\left(x\right)$ represent the KDE functions for normal and attack data, respectively. Thus, $argmin_{x}\left|KDE_{0}\left(x\right)-KDE_{1}\left(x\right)\right|$ denotes the value of *x* where $KDE_{0}\left(x\right)$ and $KDE_{1}\left(x\right)$ are equal. If frame count *N* results in the same threshold for all four attack types, the corresponding model demonstrates excellent performance across all attack types in a given environment. Consequently, *N* was varied from 15 to 64, and the thresholds were determined for all four attack types. The results are shown in Figure 9.

The threshold increases as *N* increases, and there are 17 values of *N* for which the thresholds are the same across all four attack types. To determine the optimal frame count among these 17 values, this study adopted the sum of the probability of misclassifying normal data as attack data and the probability of misclassifying attack data as normal data as an error rate estimation (ERE). A model with an *N* that minimizes the ERE is expected to exhibit superior performance because it reduces the likelihood of misclassification. The equation used to calculate the ERE is as follows:

(8) $$ERE=\frac{\int_{L_{Th}}^{\infty }KDE_{0}\left(x\right)\;dx}{\int_{-\infty }^{\infty }KDE_{0}\left(x\right)\;dx}+\frac{\int_{-\infty }^{L_{Th}}KDE_{1}\left(x\right)\;dx}{\int_{-\infty }^{\infty }KDE_{1}\left(x\right)\;dx}$$

Here, $L_{Th}$ represents the threshold calculated using Equation (7). The ratio $\int_{L_{Th}}^{\infty }KDE_{0}\left(x\right)\;dx$ to $\int_{-\infty }^{\infty }KDE_{0}\left(x\right)\;dx$ indicates the probability of misclassifying normal data as an attack. Similarly, the ratio of $\int_{-\infty }^{L_{Th}}KDE_{1}\left(x\right)\;dx$ to $\int_{-\infty }^{\infty }KDE_{1}\left(x\right)\;dx$ represents the probability of misclassifying attack data as normal. Therefore, the sum of these two probabilities serves as a measure of how well normal and attack data are distinguished and provides insight into the likelihood of incorrect detection.

**Figure 10:**
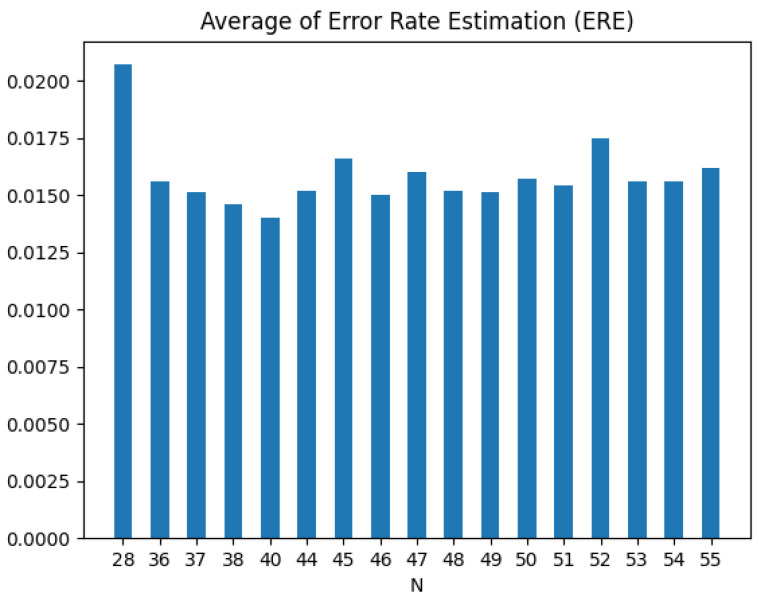
Comparison of ERE according to *N*.

For the 17 *N* values identified earlier, the ERE values for the four attack types were calculated using Equation (8), and the averaged values were obtained. The results are shown in Figure 10.

The analysis indicated that the smallest error rate was achieved when *N* = 40. Therefore, it is concluded that when *N* is 40, the model exhibits superior performance by having the same threshold across all four attack types while minimizing the probabilities of misclassification.

### 3.5. Hardware Implementation

**Figure 11:**
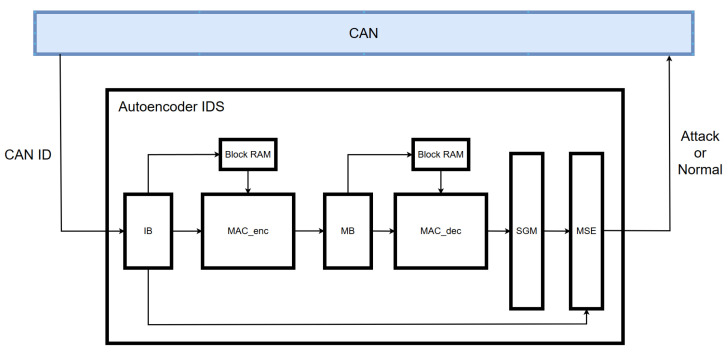
Block diagram of proposed IDS.

The block diagram of the proposed IDS implemented in hardware is shown in Figure 11, and its operational flow is described as follows:

1. The 11-bit CAN ID is received and expanded to 29 bits using a zero-padding technique.
2. The transformed 29-bit CAN ID is sent bit by bit to the MAC_enc module and simultaneously transferred to the MSE module for later loss computation.
3. In the MAC_enc module, each input bit received from the input buffer is multiplied with a 16-bit weight retrieved from Block RAM and then accumulated.
4. Once the operations for 40 CAN IDs are completed, the accumulated value is passed to the middle buffer module.
5. The middle buffer module adds a bias term to the received value, applies the ReLU function, and forwards the result to the MAC_dec module.
6. In the MAC_dec module, the 16-bit value is multiplied by a 16-bit weight retrieved from Block RAM and summed.
7. After all computations are completed, a bias term is added and the result is forwarded to the sigmoid module.
8. The sigmoid module applies an approximated sigmoid function and sends the output to the MSE module.
9. The MSE module calculates the loss using the received output and the originally stored input value based on the mean squared error method.
10. Finally, the computed loss is compared with a predefined threshold to determine whether an attack is present.

#### 3.5.1. Parameter

**Figure 12:**
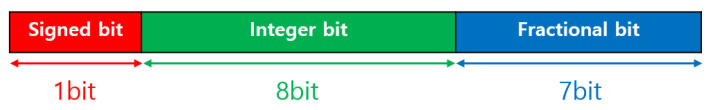
16-bit fixed point.

In this study, the proposed software model was implemented in hardware by quantizing all parameters using a 16-bit fixed-point representation consisting of a 1-bit sign, 8-bit integer, and 7-bit fractional component. This quantization scheme is illustrated in Figure 12.

From a hardware design perspective, fixed-point arithmetic requires fewer logic gates and consumes less power compared to floating-point operations. Furthermore, it eliminates the need for normalization, thereby reducing hardware resource usage. Accordingly, in this study, the weights and biases extracted from the software model were quantized into 16-bit fixed-point format and applied to the hardware implementation.

#### 3.5.2. PLAN Sigmoid

To approximate the sigmoid activation function in hardware, this study adopts a simplified piecewise linear method known as PLAN (Piecewise Linear Approximation of Nonlinearity). The standard sigmoid function is defined as follows:

(9) $$\sigma \left(x\right)=\frac{1}{1+e^{-x}}$$

This function involves exponential and division operations, making it difficult to implement directly in hardware. Although a look-up table (LUT) could be used, it would lead to excessive resource consumption. Therefore, this study adopts a piecewise linear approximation that preserves the general shape of the sigmoid function while reducing computational overhead. The input range is divided into four segments based on the absolute value, and a separate linear expression is applied to each. The specific functions for each segment are summarized in Table 3.

#### 3.5.3. Hardware Verification Environment

**Figure 13:**
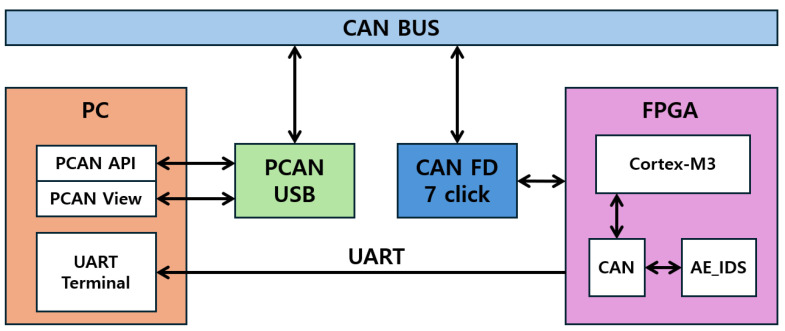
Block diagram of the FPGA verification environment.

**Figure 14:**
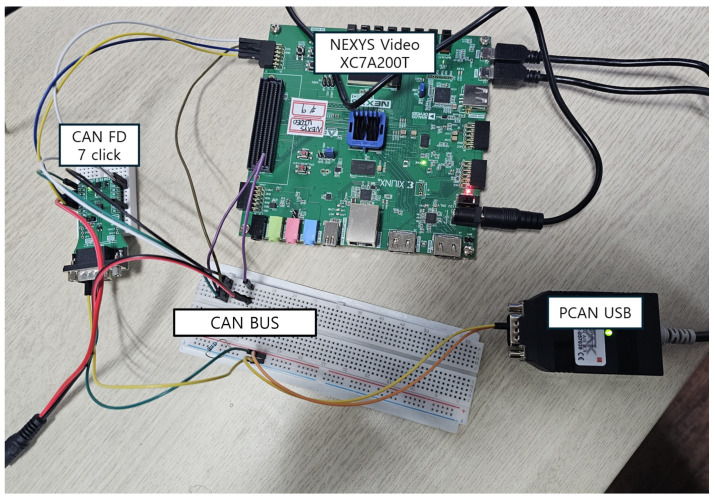
Actual FPGA verification environment.

To validate the proposed IDS on an FPGA, a verification environment was constructed as illustrated in Figure 13. An ARM Cortex-M3 processor was integrated into the FPGA to interface with the CAN module, and the designed IDS module was connected to the CAN module. A CAN FD 7 Click transceiver chip was employed to connect the FPGA to the CAN bus. To transmit CAN messages from a PC, a PCAN-USB device was placed between the PC and the CAN bus, and data transmission was performed using a Python 3.9-based PCAN API. The PCAN-View program was used to monitor the communication process and ensure data integrity. Finally, the output from the IDS module was transmitted to the PC via the FPGA’s UART interface for storage and comparison. The practical implementation of this setup is shown in Figure 14.

## 4. Results

### 4.1. Performance Metrics

In this study, the performance evaluation was conducted using four metrics: accuracy, precision, recall, and F1-score. The following section describes these metrics.

- **Accuracy**: Accuracy is the proportion of correctly classified instances out of all predictions. It evaluates how well the model classifies the entire dataset.
- **Precision**: Precision indicates the proportion of cases predicted as attacks that are actually attacks. It assesses the ability of the model to minimize false positive predictions for attacks.
- **Recall**: Recall refers to the proportion of actual attack cases that the model correctly predicts as attacks. It is particularly useful in scenarios where minimizing false negative predictions is crucial.
- **F1-Score**: F1-score represents the harmonic mean of precision and recall and evaluates the balance between these two metrics.

The four metrics are expressed as follows:

(10) $$\mathrm{Accuracy}=\frac{\mathrm{TP}+\mathrm{TN}}{\mathrm{TP}+\mathrm{TN}+\mathrm{FP}+\mathrm{FN}}$$

(11) $$\mathrm{Precision}=\frac{\mathrm{TP}}{\mathrm{TP}+\mathrm{FP}}$$

(12) $$\mathrm{Recall}=\frac{\mathrm{TP}}{\mathrm{TP}+\mathrm{FN}}$$

(13) $$F1-\mathrm{Score}=\frac{2\times \mathrm{Precision}\times \mathrm{Recall}}{\mathrm{Precision}+\mathrm{Recall}}$$

Here, true positives (TP) represent instances where attacks were correctly classified as attacks and true negatives (TN) indicate cases where normal data were correctly classified as normal. False positives (FP) refer to instances where normal data are incorrectly classified as attacks and false negatives (FN) denote cases where attacks are incorrectly classified as normal.

### 4.2. Experimental Results

In this study, the proposed IDS was evaluated using attack datasets that were entirely excluded from both the model training and the processes of threshold selection and frame count selection. The performance of the proposed model for each attack type was evaluated using the four aforementioned metrics, and the results are presented in Table 4.

In addition, the memory requirements and size of the model were compared with other IDS models that were not implemented in hardware by calculating the number of parameters and floating-point operations (FLOPs). The number of parameters is directly associated with the memory requirements of the model and is considered a critical metric in lightweight model designs. FLOPs represent the computational complexity of the model, providing a quantitative evaluation of the computation required for the model to perform a specific task, that is, the model size. We employed a simple autoencoder model consisting of two dense layers, resulting in a small model size that can be quantified by calculating the FLOPs as follows:

(14) FLOPs=(2A−1)×B,

where *A* represents the number of input nodes and *B* represents the number of output nodes. The reason for multiplying (2A−1) by *B* is that, in the case of fully connected layer operations, each output node involves *A* multiplication operations and (A−1) addition operations. In other words, (2A−1) operations are required for each output node and this process is repeated for all *B* output nodes.

For the proposed model, the FLOPs calculated using (13) and the number of parameters were compared with those of other IDS models. The results are summarized in Table 5.

Table 4 and Table 5 compare the performance of the proposed model with those of existing supervised learning-based IDSs [7] and unsupervised learning-based IDSs [8,9,10].

The deep convolutional neural network (DCNN) model [7] demonstrates superior performance compared to other models. However, because it employs a supervised learning approach for the IDS, it cannot detect attacks that have not been included in the training data. Furthermore, analysis of the FLOPs and the number of parameters revealed that the model was significantly larger than its counterparts, which constituted a notable drawback. The results of the performance comparison of the proposed model with similar unsupervised learning-based IDSs is as follows. The proposed model outperforms the GAN-based IDS (GIDS) model [8] in all metrics except for recall in detecting DoS and fuzzy attacks. Similarly, when compared with the iForest-based IDS [9], the proposed model achieved better performance in most metrics, except recall. The NovelADS model [10], on the other hand, demonstrates superior overall performance compared to the proposed model. However, NovelADS requires different thresholds for each attack type, which necessitates a separate model configuration for each type. This approach significantly increases the hardware size and software computation requirements, as listed in Table 5, because the model must be reconfigured with unique thresholds for each attack type during its deployment in a vehicle. Considering this, the proposed model, which uses a single threshold and has fewer FLOPs and fewer parameters, is more suitable for environments with multiple attack types, owing to its efficiency and adaptability in attack detection.

In addition, the results obtained from the hardware implementation were compared with the software performance of the proposed model using the previously mentioned metrics: accuracy, precision, recall, and F1-score. The comparison is summarized in Table 6.

While the original software implementation used 32-bit floating-point parameters, the hardware version employed 16-bit fixed-point representations, resulting in slight variations in performance. However, unlike conventional deep learning models where the final decision is derived directly from the model’s output, the IDS proposed in this study determines attacks based on whether the model’s loss exceeds a predefined threshold. This structural distinction makes the proposed IDS less susceptible to performance degradation during hardware implementation. As shown in Table 6, the proposed IDS demonstrated a slight overall improvement in performance for three out of four attack types—excluding the fuzzy attack—after hardware deployment.

Table 7 presents the performance comparison between the proposed IDS implemented in hardware and other FPGA-based IDS approaches.

To compare the proposed IDS with other IDS approaches implemented in hardware using an FPGA, several hardware metrics were considered, including FPGA device type, the number of LUTs and flip-flops, BRAM and URAM size, power consumption, and latency. In terms of LUT usage, the proposed IDS reduced resource consumption by approximately 84% compared to QMLP and 72% compared to BNN. For flip-flops, it achieved reductions of about 85% and 81% relative to QMLP and BNN, respectively. Regarding power consumption, the proposed IDS showed a decrease of approximately 93% compared to QMLP and 89% compared to BNN. Additionally, the latency was reduced by an average of 0.18 ms compared to both IDS models.

### 4.3. Discussion and Limitation

The adaptive autoencoder-based IDS proposed in this study learns only the CAN frame IDs. However, instead of treating each ID independently, it learns sequences composed of *N* consecutive frames. This enables the IDS to capture temporal variations across entire sequences, allowing it to effectively detect not only DoS and fuzzy attacks but also spoofing attacks that involve subtle changes in payloads.

Unsupervised learning methods, including our proposed approach, are generally effective in detecting previously unseen attack types and tend to exhibit somewhat lower performance compared to supervised learning methods when dealing with well-known and previously observed attacks. As one of the performance metrics, the precision metric represents the proportion of correctly identified attacks among all instances classified as attacks, with a higher precision indicating a lower likelihood of misclassifying normal messages as malicious. Such false positives may lead to message drops or alert spam in real vehicular environments. Although the proposed model achieved an average precision of 99.2%, a 0.8% false positive rate remains, which could potentially undermine system reliability. Therefore, future research should aim to further improve precision while maintaining other performance metrics such as recall and F1-score in order to minimize false positives and meet the stringent reliability requirements of automotive safety systems.

## 5. Conclusions

This study proposes an adaptive CAN IDS using an autoencoder model and a KDE function. As an unsupervised learning model, the autoencoder is trained exclusively on normal data. This characteristic allows the model to identify untrained attacks unlike supervised learning models. The trained autoencoder model distinguishes between normal and attack data based on the reconstruction loss, using a predefined threshold. In this study, a KDE function was applied to the loss values obtained from attack data that were not included in the test dataset, allowing a single threshold to be established for all four types of attacks. This demonstrates that a single model can effectively detect multiple types of attacks. Additionally, by defining and comparing the ERE, the optimal number of frames for effectively distinguishing between normal and attack data was determined. The proposed model was tested using four types of attack data. The results showed average accuracy, precision, recall, and F1-score of 99.19%, 99.16%, 99.13%, and 99.14%, respectively. These findings demonstrate that the proposed model outperforms existing unsupervised learning-based IDS models. For hardware implementation, the parameters were quantized from 32-bit floating-point to 16-bit fixed-point representation, and the PLAN sigmoid approximation was adopted during the design process. To ensure reliable validation, the FPGA board was configured with a Cortex-M3 processor, a CAN module, and the fully designed hardware model. A test environment was established by connecting a CAN transceiver chip, a PCAN interface, and the CAN bus, enabling the transmission of real-time CAN messages. Through this setup, the proposed IDS was verified to operate correctly and demonstrated reduced hardware resource usage compared to other IDS implementations. After hardware deployment, the system was tested using four types of attack data. The resulting performance metrics showed an average accuracy of 99.21%, precision of 99.18%, recall of 99.14%, and F1-score of 99.16%. Future work will focus on improving the precision metric to 100% while maintaining the other performance indicators, with the goal of minimizing false positives and meeting the stringent reliability standards required for automotive safety systems.

## Figures and Tables

**Table 1:** Limitations of existing CAN IDS approaches and enhancements in the proposed model.

IDS Model	Limitation	Our Model
Supervised Learning	Can detect only attacks used in training	Can detect attacks not used in training
GAN	Lower performance compared to supervised models	Comparable performance to supervised models
iForest	Lower performance compared to supervised models	Comparable performance to supervised models
NovelADS	Requires separate thresholds for each attack type	Uses a single threshold for all attack types
QMLP	High hardware resource consumption	Reduced hardware resource usage through lightweight design
BNN	High hardware resource consumption	Reduced hardware resource usage through lightweight design

**Table 2:** Overview of car hacking dataset [30].

Data Type	# of Total Frame	# of Normal Frame	# of Attack Frame
Normal	988,987	988,987	-
DoS attack	3,665,771	3,078,250	587,521
Fuzzy attack	3,838,860	3,347,013	491,847
Gear attack	4,443,142	3,845,890	597,252
RPM attack	4,621,702	3,966,805	654,897

**Table 3:** Piecewise definition of the PLAN Sigmoid function.

Condition	Operation
|X|≥5	Y=1
2.375≤|X|<5	Y=0.03125×|X|+0.84375
1≤|X|<2.375	Y=0.125×|X|+0.625
0≤|X|<1	Y=0.25×|X|+0.5
X<0	Y=1−Y

**Table 4:** Comparison of model performance based on different training features.

Attack Type	Learning Method	Detection Model	Accuracy	Precision	Recall	F1-Score
	Supervised	DCNN	99.97	100	99.89	99.95
		GIDS	97.90	96.80	99.60	98.18
DoS attack	Unsupervised	iForest	-	-	-	-
		NovelADS	-	99.97	99.91	99.94
		**Our Model**	**98.87**	**98.19**	**98.98**	**98.58**
	Supervised	DCNN	99.82	99.95	99.65	99.80
		GIDS	98.00	97.30	99.50	98.39
Fuzzy attack	Unsupervised	iForest	99.29	95.07	99.93	97.44
		NovelADS	-	99.99	100	100
		**Our Model**	**99.48**	**99.54**	**99.43**	**99.48**
	Supervised	DCNN	99.95	99.99	99.89	99.94
		GIDS	96.20	98.10	96.50	97.29
Gear attack	Unsupervised	iForest	99.24	94.79	100	97.33
		NovelADS	-	99.89	99.93	99.91
		**Our Model**	**99.16**	**99.40**	**99.00**	**99.20**
	Supervised	DCNN	99.97	99.99	99.94	99.96
		GIDS	98.00	98.30	99.00	98.65
RPM attack	Unsupervised	iForest	99.85	98.97	100	99.48
		NovelADS	-	99.91	99.90	99.91
		**Our Model**	**99.25**	**99.49**	**99.11**	**99.30**

**Table 5:** Comparison of model performance using FLOPs and parameters.

Model	FLOPs	Parameters
DCNN	100.13 M	1.71 M
GIDS	1.59 M	1.52 M
NovelADS	36.46 M	0.90 M
**Our Model**	**0.30 M**	**0.15 M**

**Table 6:** Performance comparison between the original software model and the hardware implementation.

Attack Type	IDS Type	Accuracy	Precision	Recall	F1-Score
DoS attack	Software	98.87	98.19	98.98	98.58
	Hardware	98.93	98.34	98.98	98.66
Fuzzy attack	Software	99.48	99.54	99.43	99.48
	Hardware	99.40	99.47	99.34	99.41
Gear attack	Software	99.16	99.40	99.00	99.20
	Hardware	99.17	99.37	99.04	99.21
RPM attack	Software	99.25	99.49	99.11	99.30
	Hardware	99.32	99.55	99.18	99.37

**Table 7:** Performance comparison between the proposed IDS and other FPGA-based IDSs.

Metric	QMLP-IDS	BNN-IDS	Proposed IDS
FPGA Device	ZCU104 XCZU7EV	Zedboard XC7Z020	**Nexys Video XC7A200T**
LUT	56,733	33,224	**9223**
Flip Flop	72,146	54,175	**10,472**
BRAM (Mb)	3.06	4.85	**2.39**
URAM (Mb)	6.75	0	**0**
Power (W)	3.76	2.29	**0.25**
Latency (ms)	0.24	0.26	**0.07**

## Data Availability

https://ocslab.hksecurity.net/Datasets/car-hacking-dataset (accessed on 14 May 2025).
